# Levenshtein error-correcting barcodes for multiplexed DNA sequencing

**DOI:** 10.1186/1471-2105-14-272

**Published:** 2013-09-11

**Authors:** Tilo Buschmann, Leonid V Bystrykh

**Affiliations:** 1 Institute for Medical Informatics and Biometry (IMB), Faculty of Medicine Carl Gustav Carus, Dresden University of Technology, Dresden, Germany; 2 Laboratory of Ageing Biology and Stem Cells, European Research Institute for the Biology of Ageing, University Medical Center Groningen, University of Groningen, Groningen, The Netherlands

## Abstract

**Background:**

High-throughput sequencing technologies are improving in quality, capacity and costs, providing versatile applications in DNA and RNA research. For small genomes or fraction of larger genomes, DNA samples can be mixed and loaded together on the same sequencing track. This so-called multiplexing approach relies on a specific DNA tag or barcode that is attached to the sequencing or amplification primer and hence appears at the beginning of the sequence in every read. After sequencing, each sample read is identified on the basis of the respective barcode sequence.

Alterations of DNA barcodes during synthesis, primer ligation, DNA amplification, or sequencing may lead to incorrect sample identification unless the error is revealed and corrected. This can be accomplished by implementing error correcting algorithms and codes. This barcoding strategy increases the total number of correctly identified samples, thus improving overall sequencing efficiency. Two popular sets of error-correcting codes are Hamming codes and Levenshtein codes.

**Result:**

Levenshtein codes operate only on words of known length. Since a DNA sequence with an embedded barcode is essentially one continuous long word, application of the classical Levenshtein algorithm is problematic. In this paper we demonstrate the decreased error correction capability of Levenshtein codes in a DNA context and suggest an adaptation of Levenshtein codes that is proven of efficiently correcting nucleotide errors in DNA sequences. In our adaption we take the DNA context into account and redefine the word length whenever an insertion or deletion is revealed. In simulations we show the superior error correction capability of the new method compared to traditional Levenshtein and Hamming based codes in the presence of multiple errors.

**Conclusion:**

We present an adaptation of Levenshtein codes to DNA contexts capable of correction of a pre-defined number of insertion, deletion, and substitution mutations. Our improved method is additionally capable of recovering the new length of the corrupted codeword and of correcting on average more random mutations than traditional Levenshtein or Hamming codes.

As part of this work we prepared software for the flexible generation of DNA codes based on our new approach. To adapt codes to specific experimental conditions, the user can customize sequence filtering, the number of correctable mutations and barcode length for highest performance.

## Background

High-throughput sequencing is an increasingly popular technique due to steadily improving sequencing capacity and decreasing costs. Since modern machines are (at the time of writing this manuscript) capable of generating up to 8 ∗ 10^9^ base pairs (8 Gbp) total read length in one lane, it might exceed required capacity for many research protocols focused on smaller scale sequencing applications, for instance those focused on selective DNA sampling for SNP analysis [1,2], miRNA expression profiling [3], cellular barcoding [4], profiling repeated elements [5] and retroviral vector integration sites in the genome [6], as well as complete sequencing of microbial [7] and other small genomes [8].

In such cases many samples are combined in a single batch and sequenced as one sample. Using this multiplexed format, specific sample tags, also called barcodes, are added to the amplification or sequencing primer to discriminate all sub-samples in the mixture. After sequencing, reads can be identified by reading barcodes, allowing the sorting and separating of all sequence reads into original samples. The protocol is efficient as long as barcodes can be read robustly [9].

It is known, however, that multiple errors can occur with DNA sequencing due to defects in primer synthesis, the ligation process, sample pre-amplification, and finally sequencing. These errors can be either nucleotide substitutions or small insertions and deletions [10]. In addition to common sources of error, some sequencing platforms show elevated error rates in specific situations, such as indels of identical bases in Roche 454 Pyrosequencing [11] or random indels in PacBio sequencing technology [12]. Although any randomly picked synthetic nucleotide sequence can be used as a barcode, this approach is problematic because all basic parameters of the corresponding oligonucleotide, namely minimal distance, GC content, sequence redundancy etc. cannot be properly controlled [13].

In recent years several papers were published attempting to utilize general coding theory of binary error-correcting codes. The major advantage of those codes over “naive” tags is the possibility to detect and correct a limited number of errors. In addition they also ensure a constant minimal distance. Other parameters, such as GC content and sequence redundancy, are generally more uniform in error-correcting codes than in randomly generated tags.

Probably the first attempt to create a systematic error-correcting code for DNA barcodes was made by Hamady et al. [7], based on the original Hamming binary code [14,15]. The authors adapted Hamming codes for a DNA context by representing each DNA base by two consecutive binary digits. Although being popular for a while, this barcode was later found to be flawed [13,16]: in a proposed configuration one third of all single errors occurring at the DNA level caused 2 bit changes (2 errors) in the code. By definition those 2 bit errors could not be corrected. As an alternative, Krishnan et al. proposed to use binary, linear error-correcting codes for DNA barcoding applications [16]. Those codes provide larger minimal distance and better error-correcting capacity. This allowed correction of DNA errors even if there were 2-bit errors in the code. Recently one of us proposed to adapt Hamming binary code to the DNA quaternary metric, thus preserving minimal distance and capability to correct single errors on the DNA level [13]. Both applications [13,16], however, were dedicated to the linear perfect codes capable of correcting nucleotide substitutions only. As indicated above, insertions and deletions (indels) might be a persistent problem for at least some sequencing platforms. Therefore it is very important to design a code resistant to this type of error as well. In this manuscript we provide a code, which we call the Sequence-Levenshtein code, capable of correcting all types of errors, including insertions and deletions. This code largely follows ideas from the Levenshtein code [17]. Unlike previous attempts at adapting Levenshtein [18], it is specifically designed for the DNA context. As a consequence it shows significant improvements in recovering errors in DNA sequence compared to other codes of the same kind.

## Method

### Barcode preparation

Barcodes were constructed as DNA sequences of fixed length *n* from the 4 different bases. Here, we encoded DNA bases A, C, G, T as numbers 0, 1, 2, and 3 in a quaternary alphabet and therefore avoided the binary-quaternary conversions used by others [7,16]. The number of all possible combinations, and therefore the size of the maximum barcode set was 4^*n*^, e.g. an unfiltered 8-mer barcode set could have been used for 4^8^ = 65536 unique samples. For the calculation of maximal set sizes of barcodes of length *n*, we initially generated the full set of all possible barcodes with our custom software written in Java. This initial barcode set was then filtered to exclude barcodes with GC-content of less than 40% or more than 60%, perfect self-complementation, or more than two sequential repetitions of the same base.

### Error-correcting codes

Error-correcting DNA barcode sets were constructed using only a subset of the 4^*n*^ maximal combinations, while carefully meeting some specific error-correcting properties. Commonly, this subset is called *code* and the individual barcodes in the set are called *codewords*.

**Figure 1:**
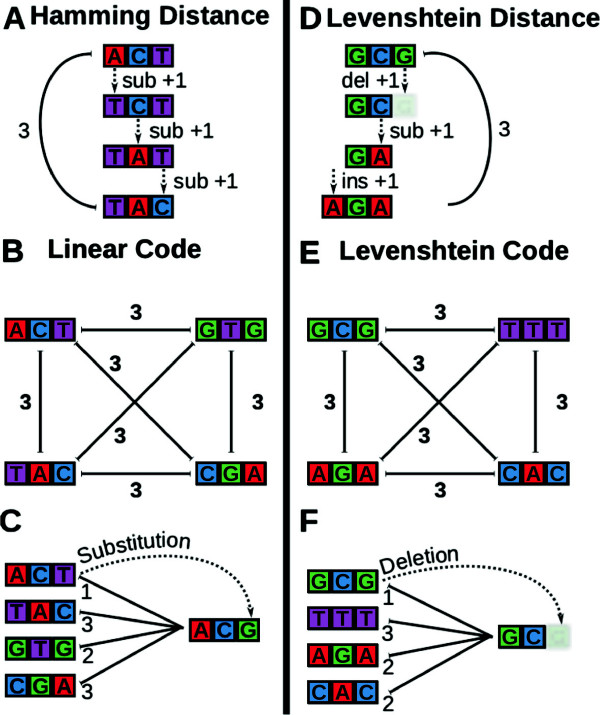
**Barcode correction using Hamming and Levenshtein distances. (A)** Hamming distance between two example codewords. **(B)** Example linear code of four codewords with the minimal Hamming distance $d_{H}^{\text{min}}=3$. **(C)** A mutation is corrected on the basis of the minimal distance between barcodes and mutated sequence. **(D,E,F)** The same for Levenshtein distance and an example Levenshtein code with the minimum Levenshtein distance $d_{L}^{\text{min}}=3$.

A very popular code for the correction of substitution errors is based on the idea of *linear codes* (e.g. Hamming codes [14,15] or Reed-Solomon codes [19]). This type of code consists only of codewords that differ in at least three positions from each other (called the *Minimum Hamming Distance*, denoted as $d_{H}^{\text{min}}$). Figures 1(A-C) depict the Hamming distance and its application in DNA context.

Figure 1(B) gives an example of a linear code that has a minimal Hamming distance of 3 and corrects 1 substitution error. A substitution error and its correction is shown in Figure 1(C): The barcode “ACT” mutates at position 3 and the base “T” became substituted with the base “G”. The Hamming distance to the original barcode “ACT” is 1, while it is greater for all other barcodes of this linear code. Therefore correct decoding and identification of the original barcode is possible. In general, more substitution errors can be corrected by constructing codes with a larger minimal distance between codewords. To correct *k* errors, the minimum Hamming distance $d_{H}^{\text{min}}$ of the codewords needs to be at least 2 ∗ *k* + 1.

As in the case of linear codes, Levenshtein-based codes guarantee a specific minimum distance $d_{L}^{\text{min}}$ between any codewords [17]. The difference is in the distance definition: Levenshtein based codes also include insertions and deletions that need to occur to transform one word to another word, as depicted in Figure 1(D). Levenshtein-based codes consisting of codewords with a minimum Levenshtein distance $d_{L}^{\text{min}}>2*k+1$ can correct *k* insertions, deletions, and substitutions. Figure 1(E) depicts an example code with $d_{L}^{\text{min}}=3$ that corrects 1 insertion, deletion, or substitution error when not in context of other DNA. Figure 1(F) shows such a correction: The last base of barcode “GCG” becomes deleted and is read as “GC”. The Levenshtein distance to the original barcode “GCG” is 1, while it is greater for all other barcodes of this Levenshtein code. Therefore correct decoding and identification of the original barcode is possible.

For the purpose of this paper, the *error-correction capability* of a code is the number and types of errors that a code (per design) guarantees to correct in a specific scenario. The actual error-correction capabilities in realistic scenarios (e.g. biological experiments, PCR and sequencing data) will be studied separately.

### Sequence-Levenshtein distance

We adapted the Levenshtein distances in such a way that the DNA context is taken into account and the length of the new mutated barcode in the sequence read is correctly identified. In the worst case, any barcode embedded in the sequence read will be surrounded by the sample sequence such that it decreases its distance to other sequences in the set.

The Sequence-Levenshtein distance between two arbitrary words A and B is the minimum number of the following three operations: 

• Substitutions

• Deletions

• Insertions

which results in word Ā, finalized by applying one of the following operations exactly once: 

• Truncating Ā to match the length of B

• Elongating Ā to match the length and bases of B

The latter two operations do not increase the distance between A and B. It follows, that the distance between A and B is 0 if A is a prefix of B (and vice versa). For the purpose of this distance metric, we define in this case A to be equal to B.

### Barcode computation

There is no systematic calculation rule for the classic Levenshtein code and codes based on our Sequence-Levenshtein distance. A generation of distance-based codes by an exhaustive search of the set of all possible subsets has two computational bottlenecks that have to be addressed: Firstly, the number of all subsets grows exponential with the length of the codewords and therefore the enumeration of these subsets is prohibitively inefficient. Secondly the distance between any two codewords has to be calculated at least once, making $\frac{4^{2n}}{2}-4^{n}$ calculations necessary. Distances need to be calculated repeatedly if the complete distance matrix cannot be held in memory.

We therefore generated codes heuristically with a so-called *greedy closure evolutionary* algorithm first described for this application by Ashlock et al. [20,21]. Here, we initialized our code set with a small number (2-4) of random barcodes that fulfill the distance requirement (the so-called *seed*). We then walked through all eligible barcodes in lexicographical order and added the tested barcode to the code set if its distance was at least 2 ∗ *k* + 1 to every other barcode that was already in the code set. Using an evolutionary approach (in the computational sense), we tried a large number of different seeds or altered very successful seeds to find the seed giving the best, i.e. largest code set. Among other heuristic algorithms for the generation of classic Levenshtein codes, this particular method has shown the best results (Houghten *et al*[22]). The same study revealed that this method yielded nearly-optimal solutions for short codewords (*n* ≤ 5) and it reached approximately one third to one half of the upper limit of code sizes for longer codewords (5 < *n* ≤ 12) [22,23].

We also optimized the calculation of the Sequence-Levenshtein distance. We adapted the dynamic programming approach to the classical Levenshtein distance [24] and reached approximately the same performance (see Additional file 1: Supplement). Additionally, we minimized the number of operations with the approach developed by Allison (*Lazy Programming*, [25]).

### Simulations

We simulated three scenarios both with classical Levenshtein codes and modified Sequence-Levenshtein codes: 

• In *Simulation 1* the application of classical Levenshtein codes in DNA context was assessed. A large number of barcodes of the same length was generated at random, followed by a random sample sequence. Every barcode was mutated with a single random in/del/sub error and then attempted to be decoded. As the length of the received codeword was unknown, the codeword of equal length to the generated DNA barcodes was used. If decoding did not work (i.e. there was no DNA barcode with a distance of 1 to the received codeword), codewords of the length *n* - 1 and *n* + 1 were tried. If ambiguities still existed, we decided randomly.

• In *Simulation 2* the error correction capabilities of Sequence-Levenshtein codes were tested. Every code used in this manuscript was included, up to a length of 12nt for 1 and 2 correctable errors. We iterated through every possible error (1 error, respectively 2 errors; insertions, substitutions, and deletions) and decoded the resulting DNA barcode.

• In an experimental setup, more than one error might occur. Therefore, in *Simulation 3* a large number of classic Levenshtein and new Sequence-Levenshtein barcodes was simulated, where every base had a chance *p* of being mutated with equal likelihood for substitutions, insertions and deletions. Every base was equally likely to be inserted.

## Results

### Classic Levenshtein codes fail in DNA context

Levenshtein-based codes have one mandatory condition: The length of the codewords and the received words need to be known. While we know the length of the DNA barcodes because we construct them ourselves, the length of the received codeword is not available as the barcode is embedded into the DNA sequence. If the DNA barcode is shortened during processing, the first base of the sample DNA sequence takes the place of the last base of the DNA barcode. If the DNA barcode is elongated, the last base of the DNA barcode now becomes the first base of the sample DNA sequence. There is no inherent separation between DNA barcode and sample sequence to detect this change in length and thus traditional Levenshtein correction fails. To show this, we construct two codewords *c*_*A*_ and *c*_*B*_ whose Levenshtein distance is 3 but is reduced by the inference of the remaining sample DNA sequence.

**Figure 2:**
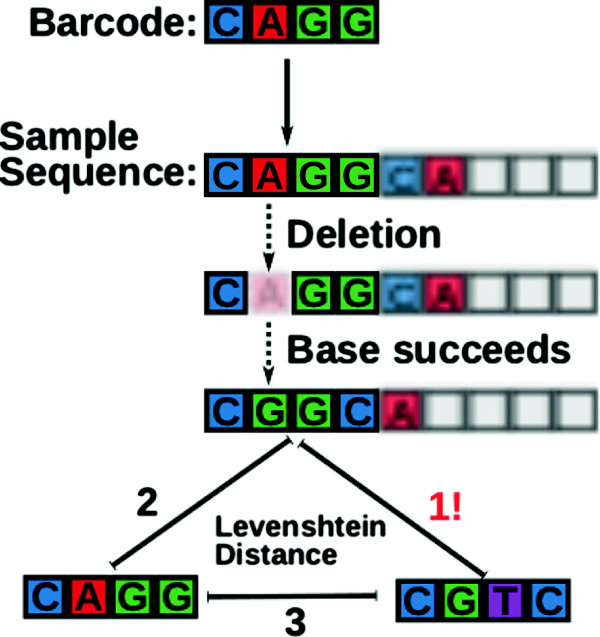
**Deficiency of Levenshtein Codes in DNA context.** Classical Levenshtein-based codes fail in DNA context as the word boundary is not decodable. Here, the original barcode “CAGG” becomes corrupted through a deletion. The new barcode “CGGC” is now closer to the wrong barcode “CGTC” on the left as opposed to the original barcode “CAGG” on the right.

We construct the codewords *c*_*A*_ = “CAGG” and *c*_*B*_ = “CGTC” with a Levenshtein-distance *d*_*L*_ (*c*_*A*_,*c*_*B*_) = 3. In an exemplary biological experiment, *c*_*A*_ could be used as a barcode and within it could be followed by “CA” so that the whole DNA sequence reads “CAGG|CA...”. If the base “A” at the second position of *c*_*A*_ becomes deleted, the base “C” (previously on position 5) would succeed the base at position 4 so that the sequenced DNA *c*_received_ now would read: “CGGC|A...” (Figure 2). Because the deletion would remain undetected, we could try to find a correction for *c*_received_ = CGGC. Consequently, the codeword *c*_*B*_ is actually closer to the manipulated received sequence (*d*_*L*_(*c*_*B*_,*c*_received_) = 1) than codeword *c*_*A*_ (*d*_*L*_ (*c*_*A*_,*c*_received_) = 2) and there is no possibility to find the actual chain of mutations because the only criteria in correcting errors is the minimal distance. Trying to guess the real length of the corrupted barcode gives ambiguous results as Table 1 shows.

**Table 1:** Distances of the received codeword at various presumed word lengths

**Presumed**	**Presumed**	**Candidate**	
**word length**	**word boundary**	**barcodes**	
		**“CAGG”**	**“CGTC”**
3	“CGG|CA”	1	2
4	“CGGC|A”	2	1
5	“CGGCA|”	3	2

We compare two candidate barcodes “CAGG” and “CGTC” with different presumed word lengths and boundaries. Levenshtein distances for word boundaries presumed at 3 and 4 conflict and an unambiguous identification of the original used barcode is not possible.

**Figure 3:**
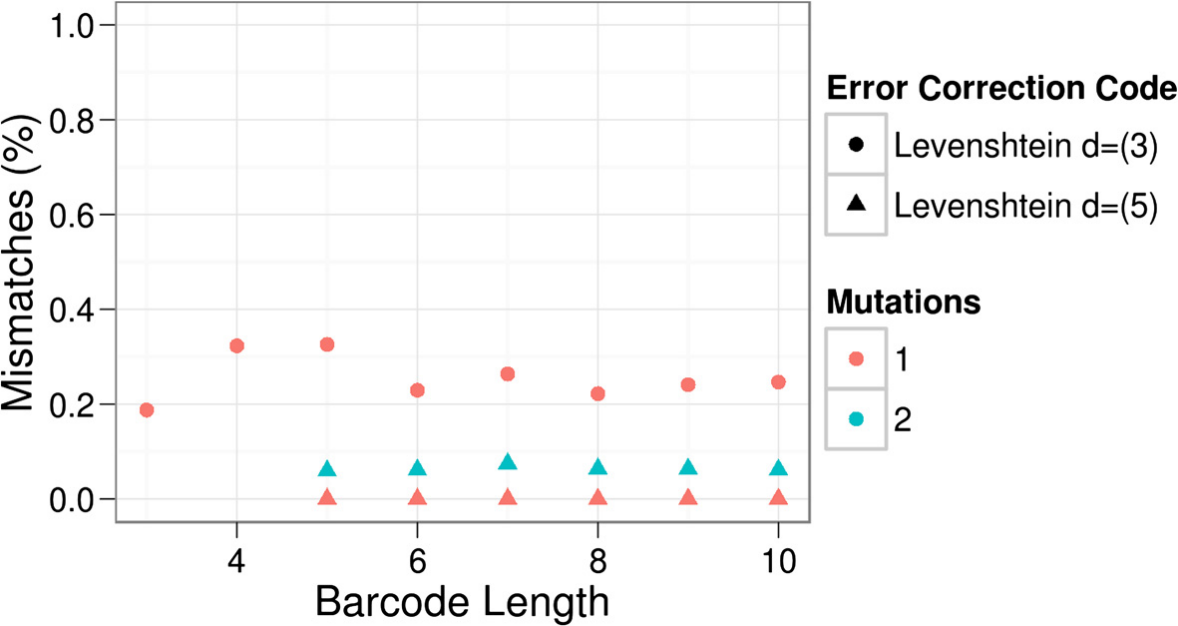
**Simulation of Levenshtein Codes in DNA context.** Levenshtein codes with a minimal distance $d_{L}^{\text{min}}=3$ failed to correct indel errors on average in 26% of the cases while Levenshtein codes with a minimal distance $d_{L}^{\text{min}}=5$ always corrected one indel error but failed to decode two indel errors in about 6.5% of the cases.

We generalized this problem in Simulation 1 (Figure 3): Barcodes based on classical Levenshtein codes with a minimal distance $d_{L}^{\text{min}}=3$ failed to correct indel errors on average in 26% of the cases (see Methods for details). This error level is very close to $\frac{1}{4}$, the probability of the adverse base to be inserted or the adverse base to be added to the barcode after a deletion. Accordingly, classical Levenshtein-based codes correctly decoded barcodes that were corrupted once if the codes have the guaranteed capability to correct two errors, but failed on average in 6.5% of two-corruption cases. This error level is explained by the probability of inserting or complementing the two random worst-case bases, which is ${\left(\frac{1}{4}\right)}^{2}=\frac{1}{16}=0.0625$.

Clarke and Ferreira previously showed that Levenshtein codes with a minimal distance $d_{L}^{\text{min}}=5$ can robustly correct at least one error in a context scenario with fixed-length decoding as applied here [26]. Henceforth, we will delineate the *guaranteed minimal error-correction capability* of Levenshtein codes specifically in DNA context under this assumption, so that Levenshtein codes with $d_{L}^{\text{min}}=5$ guarantee to correct at least one error, those with a minimal distance $d_{L}^{\text{min}}=9$ guarantee to correct at least two errors in DNA context.

### Sequence-Levenshtein distance

With the adapted Sequence-Levenshtein metric, the distance between the previously considered codewords “CAGG” and “CGTC” is now *d*_*SL*_ (“CAGG”,“CGTC”) = 2: Delete second base “A” of “CAGG” to get “CGG” and substitute third base “G” with “T” to get “CGT”. In the worst case the remaining sample sequence will start with base “C”, so that if we elongate with “C” then get “CGTC”. Therefore, “CAGG” and “CGTC” cannot be part of the same error correcting code.

The formal definition of our Sequence-Levenshtein metric allowed us to prove that it is indeed a “distance metric” (see Additional file 1: Supplement), so that codes based on this distance can correct *k* substitutions and indels in DNA context if their minimum distance is at least $d_{\text{SL}}^{\text{min}}=2*k+1$.

### Sequence-Levenshtein code example and decoding

An example of a Sequence-Levenshtein code with 4 bases for the correction of 1 error yielded 4 barcodes: “TTCC”, “ACAC”, “CGAA”, and “TAGG”. Suppose, we use “TTCC” as the barcode and the base “T” at the second position becomes deleted during sequencing. In our example, exemplary sample reads have the length *m* = 10 and the sequence read is “TCC|ATGCATA” ( 4). To decode this example, we calculate the distance between the word “TCCATGCATA” and the words “TTCC”, “ACAC”, “CGAA”, and “TAGG” with the results in Table 2. The column “operations” is the listing of the possible operations that corrupted the barcode.

**Figure 4:**
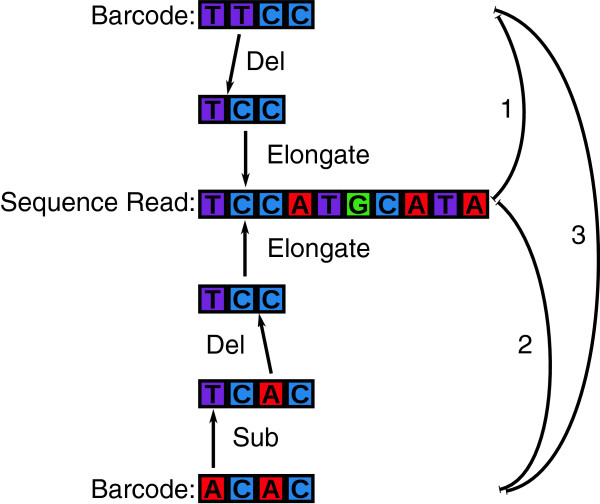
Operations in Sequence-Levenshtein distance.

**Table 2:** Example decoding results

**Candidate barcode**	**Distance to “TCCATGCATA”**	**Possible chain of operations**	**Resulting word boundary**
“TTCC”	1	del(2),elong(“ATGCATA”)	3 (“TCC|ATGCATA”)
“ACAC”	2	sub(“T”,1),del(3),elong(“ATGCATA”)	3 (“TCC|ATGCATA”)
“CGAA”	3	ins(“T”,1),sub(“C”,3),del(5),elong(“TGCATA”)	4 (“TCCA|TGCATA”)
“TAGG”	3	sub(“C”,2),sub(“C”,3),sub(“A”,4),elong(“TGCATA”)	4 (“TCCA|TGCATA”)

Table shows the results of decoding the example sequence read “TCCATGCATA” for four different candidate barcodes “TTCC”, “ACAC”, “CGAA”, and “TAGG”. The real original barcode “TTCC” has the shortest Sequence-Levenshtein distance to this sequence read and the word boundary is estimated correctly at 3.

It is apparent that the difference between the number of insertion and deletion operations is the difference between the barcode length and the starting part of the sample sequence, which allowed the identification of the starting position of the sample sequence, as shown in column four of Table 2.

### Sequence-Levenshtein codes useful for DNA applications

**Figure 5:**
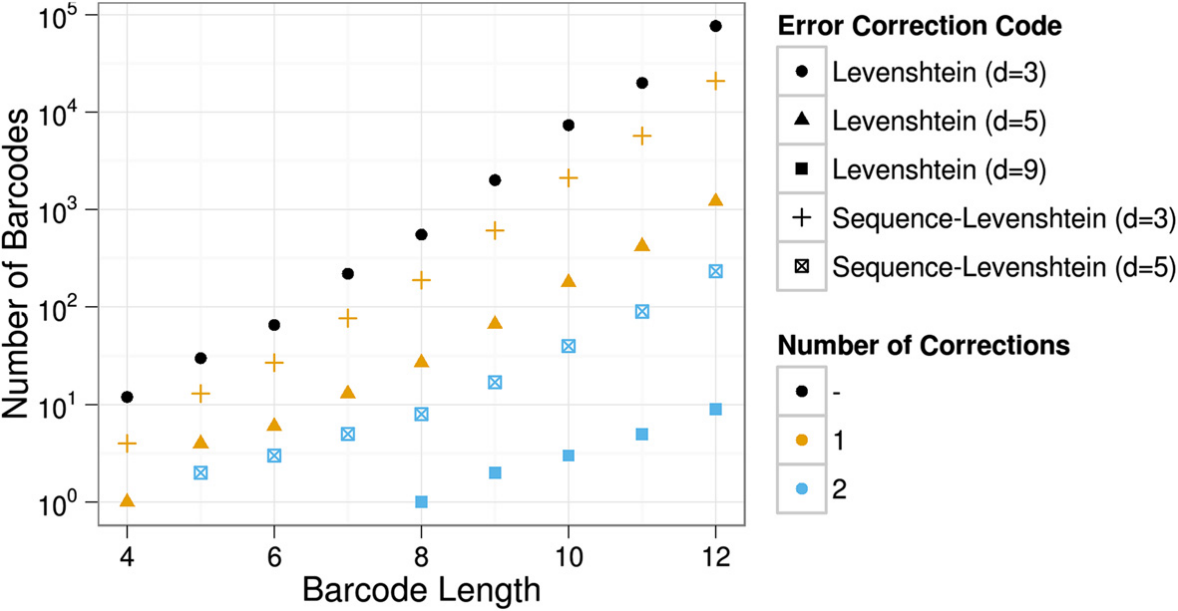
**Number of Barcodes vs Barcode Length.** Barcodes based on the Sequence-Levenshtein distance resulted in barcodes with a magnitude higher numbers then Levenshtein barcodes for the same length of the barcode and the same guaranteed minimal error correction capability. The guaranteed correction of one additional error shrunk the number of barcodes by almost two magnitudes.

We calculated and verified a number of Sequence-Levenshtein codes for different sequence lengths and compared them to codes with higher Levenshtein distance that were designed for the correction of at least this particular number of errors (the *guaranteed error correction capability* in DNA context). Figure 5 depicts the number of DNA barcodes that we generated for the correction of at least 1 or 2 insertion, deletion, and substitution errors with our Sequence-Levenshtein distance and with the classic Levenshtein distance. For comparison purposes, we also added the number of barcodes of the classical Levenshtein code with a distance $d_{L}^{\text{min}}=3$ that does not guarantee to correct at least one error reliably in DNA context.

For codewords of length 8nt, 4^8^ = 65536 possible combinations of DNA bases can be generated. Of those, 14600 met the required chemical properties as described in the Methods section. Finally, with the Sequence-Levenshtein distance a maximum barcode set of 188 elements for the correction of one error in DNA context could be generated. This is equivalent to a code rate of $\frac{{\text{log}}_{2}(188)}{{\text{log}}_{2}(65536)}\approx 0.472$. For classical Levenshtein codes, we could generate 552 barcodes, the equivalent of a code rate of $\frac{{\text{log}}_{2}(552)}{{\text{log}}_{2}(65536)}\approx 0.569$. We found that the code rate increased with barcode length for both Levenshtein and Sequence-Levenshtein based codes (see Additional 1: Figure S1).

Figure 5 shows that our modified Sequence-Levenshtein codes scaled up to more than 20,000 possible barcodes with one guaranteed correctable error. This would satisfy the needs of the most complex sample multiplexing setups. Alternatively, for a medium-sized experiment of only 48 samples, the length of the barcode did not need to exceed 7 bases (77 barcodes). Conversely, we could increase the robustness of the code to 2 correctable errors and generate 90 11-nt-long barcodes. Compared to classic Levenshtein codes, we produced one order of magnitude more barcodes for the same length and guaranteed minimal number of correctable errors.

### Simulation for correctness and the decoding rate

In Simulation 2, we simulated all possible 1 or 2 mutations for every Sequence-Levenshtein barcode used in this manuscript up to a length of 12 with the guaranteed capability to correct 1 or 2 errors and found that the original barcode could be decoded correctly in every case.

We also used this simulation to measure the speed of decoding random sequence reads with our unoptimized Java-based prototype implementation. As a general result, the number of decoded sequence reads per seconds depended on three parameters: 

• Length of the sequence read: longer was slower

• Length of barcodes: longer was slower

• Number of used barcodes: more barcodes were slower

In the slowest simulation with 20,894 12-nt-long barcodes and 14-nt-long sequence reads, we decoded 20 sequence reads per second while we decoded approximately 190,000 sequence reads per second with four 4-nt-long barcodes.

### Experimental simulation

**Figure 6:**
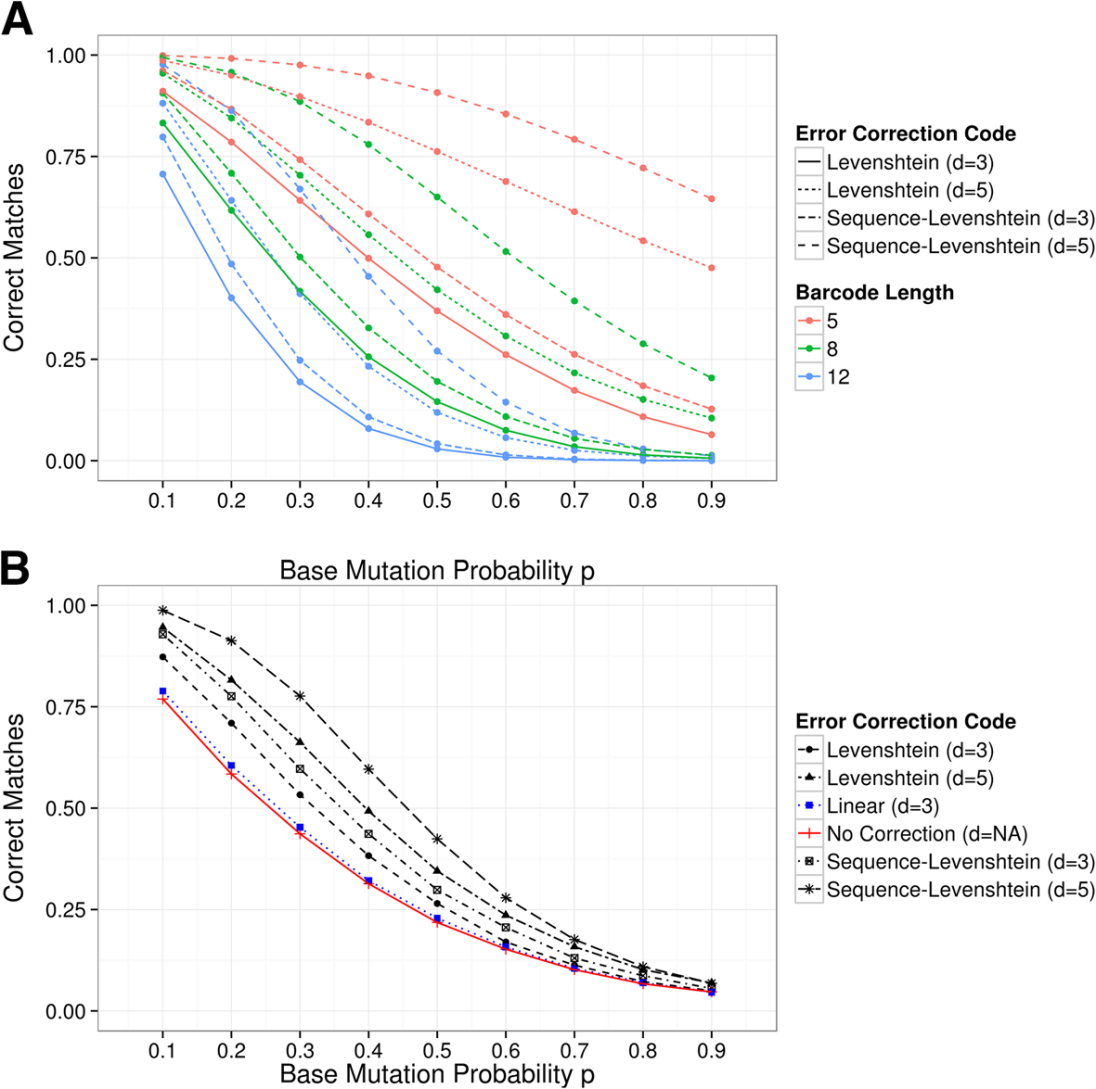
**Results of Simulation 3. (A)** Number of correct matches after decoding depending on base mutation probability rate *p* for different error correction codes and barcode lengths. **(B)** Number correct matches after decoding depending on base mutation probability rate *p* for the fixed number of 48 barcodes simulated with the smallest eligible error correction codes: Levenshtein code with $d_{L}^{\text{min}}=3$, length 6; Sequence-Levenshtein code with $d_{\text{SL}}^{\text{min}}=3$, length 7; Levenshtein code with minimum distance $d_{L}^{\text{min}}=5$, length 9; Sequence-Levenshtein code with minimum distance $d_{\text{SL}}^{\text{min}}=5$, length 11.

In Simulation 3, we analyzed the behavior and limits of Sequence-Levenshtein codes under the assumption that multiple mutations of barcodes are possible. The results are depicted in Figure 6. The theoretical expected average number of mutations *μ*_*M*_ for each barcode of length *n* and per-base mutation probability *p* was *μ*_*M*_ = *p* ∗ *n*, which we also confirmed on average in all simulation runs. As a consequence, the number of mutations in a barcode of a sequence read increased linearly with the length of the barcode, leading to a higher number of mismatches during the decoding phase (Figure 6(A)).

Sequence-Levenshtein codes have been decoded correctly at a better rate than classical Levenshtein codes of the same barcode length and the same minimal distance ($d_{\text{SL}}^{\text{min}}=d_{L}^{\text{min}}=3$ and $d_{\text{SL}}^{\text{min}}=d_{L}^{\text{min}}=5$ respectively). Furthermore, both classical Levenshtein codes and Sequence-Levenshtein codes with a higher minimal distance ($d_{\text{SL}}^{\text{min}}=5$ and $d_{L}^{\text{min}}=5$) decoded barcodes correctly more often than the same codes with a smaller minimal distance ($d_{\text{SL}}^{\text{min}}=3$ and $d_{L}^{\text{min}}=3$). Notably, although Sequence-Levenshtein codes with $d_{\text{SL}}^{\text{min}}=3$ were designed for the same guaranteed minimal number of correctable errors in DNA context as classic Levenshtein codes with $d_{L}^{\text{min}}=5$, the latter outperformed the former when a random number of mutations was considered. All these effects were more pronounced for median base mutation probabilities *p* ∈ [ 0.2,0.8].

In practice, the choice of the barcode length and the type of error correction ($d_{\text{SL}}^{\text{min}}=3$ or $d_{\text{SL}}^{\text{min}}=5$) is based on the number of samples that one wants to sequence in parallel. We therefore repeated simulation 3 on 48 barcodes from six different error correcting codes that supported this number of parallel samples: a classic Levenshtein code with $d_{L}^{\text{min}}=3$ and length 6; a classic Levenshtein code with $d_{L}^{\text{min}}=5$ and length 9; a Sequence-Levenshtein code with $d_{\text{SL}}^{\text{min}}=3$ and length 7; a Sequence-Levenshtein code with minimum distance $d_{\text{SL}}^{\text{min}}=5$ and length 11, a linear code of length 5, and finally a code of length 3 that offered no correction (see Additional  1: Table S2 for details). The result is depicted in Figure 6(B). It shows that the new Sequence-Levenshtein codes outperformed classical Levenshtein codes of the equivalent minimal distances as well as the linear code despite requiring longer barcodes. The same was true for the comparison of Sequence-Levenshtein codes with minimal distances $d_{\text{SL}}^{\text{min}}=5$ and $d_{\text{SL}}^{\text{min}}=3$. Apparently, in this case the added robustness of larger distances and the change to the classical Levenshtein distance outweighed the drawbacks of longer barcodes.

## Discussion

Historically error-correcting codes were first made in binary metric to correct program-reading errors in early type computers in the 1950s [14,27]. Levenshtein was one of the first in attempting to resolve more natural problems such as insertions and deletions [17]. Whereas computer codes were gradually evolving (in data transfer and processing, mobile, satellite communications, etc.), an application for DNA studies was far from successful. A few authors rediscovered Hamming code while making a theory of oligonucleotide design for microarrays [28,29]. This however was not implemented in commercially available microarrays. Similarly, currently available barcoded primers from, for instance, Illumina look like a random design devoid of any theoretical (error-correcting) considerations [13]. The first attempt to implement Hamming code into DNA barcode design failed due to improper binary-tertiary conversion protocol [7]. Later, this problem was resolved by adapting the Hamming concept to quaternary format [13]. Alternatively, Krishnan et al. used binary, linear error-correcting codes with longer minimal distances for DNA barcode design [16]. Whereas a noticeable progress was achieved with linear/perfect codes mentioned above, a proper application of Levenshtein codes for DNA barcodes had not yet been demonstrated. The major obstacle in these implementations was the problem of word recognition in the continuous context of DNA. As this inherent failure is not addressed in the literature on Levenshtein-based error correction in DNA barcodes (e.g. [18]), we at best assume that some form of separating sequence is used between the DNA barcode and the sample DNA, and at worst no correction of this failure was attempted. The drawback of separating sequences is obvious: they do not come with any correction ability by themselves and elongate the DNA sequence at the same time, increasing the error rate for the sample DNA. The use of separating sequences is therefore not ideal.

By simulating equally likely substitutions, deletions, and insertions we tested the robustness of Sequence-Levenshtein distance based codes. We found that the error correction of Sequence-Levenshtein barcodes was, on average, more reliable than comparable Levenshtein-based codes. Although the probabilities of mutation rates in experimental sequencing data or in biological samples might considerably deviate from equal, it very much depends on the organism and the sequencing platform. Therefore it is not easy to create a “real life” simulation of sequencing errors. In our mutation study we ignored possible differences in mutation rates solely to test as many possible mutations on as many possible DNA combinations as possible. As a result the revealed rates of successful error corrections will not necessarily correspond to those in a real sequencing data.

Sequence-Levenshtein codes can be further improved in the following ways. Firstly, as barcode libraries are often constructed only once and then reused for later experiments, it is desirable to construct barcode sets that correct *k* errors with a maximum subset that corrects *k + 1* errors. Thus, if the number of parallel processed samples in an experiment is very low, the more robust *k + 1 * subset is used. This code construction is easily achieved by modifying the evolutionary greedy search algorithm to favor barcode sets with a large robust *k + 1* subset. Secondly, not every error occurs with the same probability: some substitutions are more likely than others, e.g. DNA/RNA sequences are more likely to be altered at the end of the read than at the beginning. An advanced version of this code would therefore use probabilities of operations as a distance measure and construct codes that, while not guaranteeing error correction, will correct more errors on average with shorter barcodes.

## Conclusion

We propose a solution to the problem of the word size definition in the continuous context of DNA and a definition of a modified Levenshtein distance which we name “Sequence-Levenshtein distance”. This new distance measure takes into account the interference of appended sample sequences and the resulting shorter distances between barcodes.

This approach allows for the detection of the length of the corrupted barcode and the recovery of the start of the appended sample sequence. However, this imposes more strict rules for the selection of barcode sets eligible for error correction. We show that the application of these new barcodes decreases mismatching in multiplexing experiments considerably, increasing the robustness of experimental results. For further experimental validation and application, we provide barcode sets of different lengths and guaranteed error-correcting capabilities that will satisfy current size-needs of most experimental setups as well as software to decode sequence reads which is, in its current implementation, highly efficient.

Our Sequence-Levenshtein software package is a versatile tool to flexibly generate barcode sets of different sizes and robustness, simulate expected mismatch rates for individual next generation sequencing technologies, and decode millions of sequence reads in a short time. As such, we believe it offers a valuable research utility to the general public.

## Competing interests

Dresden University of Technology submitted a patent application for the Sequence-Levenshtein technology in Germany (application id 800228609).

## Authors’ contributions

LVB initiated the re-analysis of classical DNA codes and initiated and inspired the development of the method. TB developed the method. TB developed, ran and analysed the simulations. TB and LVB wrote the manuscript. Both authors read and approved the final manuscript.

## Supplementary Material

Additional file 1 **Supplement.** The supplement contains a proof of the metric property of the Sequence-Levenshtein distance, the dynamic programming algorithm of the Sequence-Levenshtein distance, a figure of code rates depending on barcode lengths, a table of Sequence-Levenshtein code sizes as well as an additional table describing the codes used in Simulation 3. Click here for file
