# Supplementary material for: Levenshtein error-correcting barcodes for multiplexed DNA sequencing
Source: BMC Bioinformatics. 2013 Sep 11;14:272. doi: 10.1186/1471-2105-14-272 (PMC3853030; doi:10.1186/1471-2105-14-272)
Supplement: Additional file 1 — Supplement. The supplement contains a proof of the metric property of the Sequence-Levenshtein distance, the dynamic programming algorithm of the Sequence-Levenshtein distance, a figure of code rates depending on barcode lengths, a table of Sequence-Levenshtein code sizes as well as an additional table describing the codes used in Simulation 3. [file 1471-2105-14-272-S1.pdf]

Supplement

Distance Metric Proof

A function d(x,y) is a distance metric if it observes the following conditions for all words x and y:

- $d(x,y) = 0 \iff x = y$
- $d(x,y) \geqq 0$
- $d(x,y) = d(y,x)$
- $d(x,y) \geqq d(x,z) + d(y,z)$

Proof for $d_{\mathbf{SL}}(x,y) = 0 \iff x = y$:

Three cases need to be considered:

1. Words x and y are the same sequences, i.e. they are of the same length and bases at the same position are equal. Thus, no operations are necessary to transform x into y and their distance is 0
2. Word x is a prefix of y: x is elongated to match y exactly and no other operations are necessary, in this case we consider x to be equal to y by definition
3. Word y is a prefix of x, x is truncated to match the length of y and no further operations are necessary, in this case we consider x to be equal to y by definition

■

Proof for $d_{\mathbf{SL}}(x,y) \geqq 0$

There are either no operations necessary to transform x into y ($d_{\mathrm{SL}}(x,y) = 0$) or one needs to apply substitutions, insertions, and deletions to x to transform it into y in which case $d_{\mathrm{SL}}(x,y) > 0$.■

Proof for $d_{\mathbf{SL}}(x,y) = d_{\mathbf{SL}}(y,x)$

All operations in this distance measure are symmetrical: An insertion of base B at position p (abbrv. ins(B,p)) is the reversal of deletion of base B at position p (abbrv. del(p)) and vice versa. A substitution of base B_1 with base B_2 at position p (sub(B_2,p)) is the reversal of a substitution of base B_2 with base B_1 at position p (sub(B_1,p)). Truncation (trunc()) is the reversal of the elongation (elong()) and vice versa.

The distance $d_{\mathrm{SL}}(x,y)$ can be expressed as a sequence of operations ins(), del(), sub() followed by either trunc() or elong() to match x with y, e.g.: x → sub → ins → del → trunc → y. The reversal operations sequence to transform y to x is obtained by reversing the individual substitution, deletion and insertion operations in reverse order and finalize with the reverse of the elongation or truncation operation: y → ins → del → sub → elong → x. The number of these operations is equal to the number of operations to transform x into y and therefore $d_{\mathrm{SL}}(y,x) = d_{\mathrm{SL}}(x,y)$.■

Proof for $d_{\mathbf{SL}}(x, y) \leq d_{\mathbf{SL}}(x, z) + d_{\mathbf{SL}}(z, y)$

Suppose the transformation of x to z is the result of a sequence of operations $O_{xz} =< o_{xz_1}, o_{xy_2}, \cdots, elong/trunc >$. The transformation of z to y is the sequence of operations $O_{zy} =< o_{zy_1}, o_{zy_2}, \cdots, elong/trunc >$. By the very nature of these operations, x can be transformed to y by the concatenation of both operation sequences without the elongation or truncation followed by its own truncation or elongation: $O_{xy} =< o_{xz_1}, o_{xz_2}, \cdots, o_{zy_1}, o_{zy_1}, \cdots, elong/trunc >$. The number of substitutions,deletions and insertions in O_{xy} is the sum of substitutions, deletions and insertions in O_{xz} and O_{zy} and therefore $d_{\mathrm{SL}}(x, y)$ is at most equal to $d_{\mathrm{SL}}(x, z) + d_{\mathrm{SL}}(z, y)$.■

Distance Calculation

Algorithm of distance calculation (pseudocode) using dynamical programming:

```
int function distance(Sequence sequence1, Sequence sequence2)
 set length_1 to length of sequence1
 set length_2 to length of sequence2

 declare distances[length_1+1][length_2+1]

 for i from 0 to length_1
  set distances[i][0] to i

 for j from 0 to length_2
  set distances[0][j] to j

 // Classical Levenshtein part
 for i = 1 to length_1
  for j = 1 to length_2
   set cost to 0
   if (sequence1[i-1] not equal to sequence[j-1])
    set cost to 1

   set distances[i][j] to minimum of
              distances[i-1][j-1] + cost,// Substitution
              distances[i][j-1] + 1,    // Insertion
              distances[i-1][j] + 1 // Deletion

 set min_distance to distances[length_1][length_2]


 // New Sequence-Levenshtein part

 // Truncating
 for i from 0 to length_1
  set min_distance to minimum of min_distance and distances[i][length_2]

 // Elongating
 for j from 0 to length_2
  set min_distance to minimum of min_distance and distances[length_1][j]
```

```
return min_distance
```

Code Rates

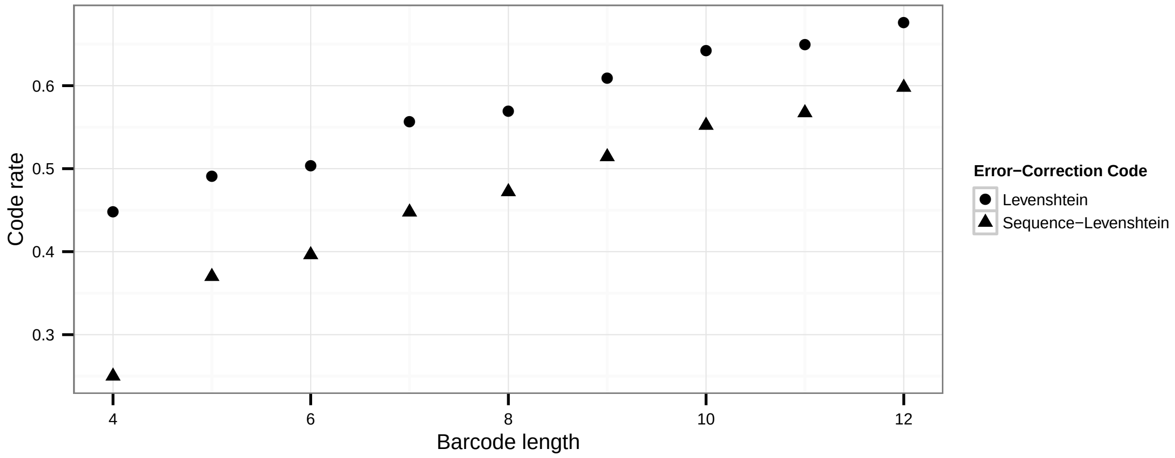

Figure S1. Code rates of Levenshtein and Sequence-Levenshtein codes depending on the lenght of codewords.

Sizes of Sequence-Levenshtein Codes

n\d	3	5
4	5	1
5	13	2
6	27	3
7	77	5
8	188	8
9	612	17
10	2123	40
11	5714	90
12	20887	232
13	-	(554)
14	-	(1583)

Table S1. Sizes of Sequence-Levenshtein Codes Code sets were filtered for biological/chemical eligibilty (c.f. Methods). We did not formally analyse or simulate barcodes of length n=13nt or n=14nt.

Codes used in Simulation 3

Of every code, a random subset of 48 barcodes was used. The details of these codes are clarified in Table S2.

Code Type	Length	Distance	Code Size
Levenshtein	6	3	66
Levenshtein	9	5	67
Sequence-Levenshtein	7	3	77
Sequence-Levenshtein	11	5	90
Linear	5	3	48
No Correction	3	NA	60

Table S2. Codes of Simulation 3
